# Evolving Concepts in Treat-to-Target Strategies for Systemic Lupus Erythematosus

**DOI:** 10.31138/mjr.290424.eci

**Published:** 2024-06-30

**Authors:** Dionysis Nikolopoulos, Maria Helena Lourenço, Roberto Depascale, Konstantinos Triantafyllias, Ioannis Parodis

**Affiliations:** 1 Division of Rheumatology, Department of Medicine Solna, Karolinska Institutet, Stockholm, Sweden,; 2 Department of Gastroenterology, Dermatology and Rheumatology, Karolinska University Hospital, Stockholm, Sweden,; 3 Department of Rheumatology, Centro Hospitalar de Lisboa Ocidental EPE, Lisbon, Portugal,; 4 Comprehensive Health Research Center (CHRC), NOVA Medical School, Universidade Nova de Lisboa, Lisbon, Portugal,; 5 Rheumatology Unit, Department of Medicine, University of Padua, Padua, Italy,; 6 Rheumatology Centre Rhineland-Palatinate, Bad Kreuznach, Germany,; 7 Department of Internal Medicine I, Division of Rheumatology and Clinical Immunology, University Medical Center of the Johannes Gutenberg University Mainz, Mainz, Germany,; 8 Department of Rheumatology, Faculty of Medicine and Health, Örebro University, Örebro, Sweden

**Keywords:** treat-to-target, systemic lupus erythematosus, remission, low disease activity, glucocorticoids

## Abstract

Systemic lupus erythematosus (SLE) is a chronic autoimmune disease that is characterised by a wide range of symptoms and a risk for irreversible organ damage, leading to increased morbidity and mortality. To improve long-term outcomes, innovative therapeutic goals have been explored, including attainment and maintenance of remission or low disease activity, with minimal use of glucocorticoids. Other goals encompass early diagnosis, potent yet less toxic therapies, appropriate glucocorticoid tapering, and better quality of life for the patients. Implementing a treat-to-target (T2T) approach involves treatment adjustments to achieve predefined objectives. Evidence from other chronic diseases, like hypertension and diabetes, supports the success of target-based approaches. In rheumatic diseases, the multitude of clinical features adds complexity to T2T strategies, but in rheumatoid arthritis, T2T has yielded improved outcomes. The application of T2T in SLE requires realistic therapeutic goals and practical tools for their measurement. International task forces have developed T2T recommendations for SLE, focusing on limiting disease activity, preventing organ damage, and minimising glucocorticoid use, while considering patients’ quality of life. Advancements in defining clinically meaningful remission and low disease activity states, coupled with promising novel therapies, have spurred progress in the management of SLE.

## INTRODUCTION

Systemic lupus erythematosus (SLE) is a chronic autoimmune condition that necessitates life-long treatment.^1^ The disease gives rise to a wide range of symptoms, which frequently serve as the primary catalysts for medical interventions. However, disease activity is not in its entirety detectable through recognisable symptoms.^2^ Although significant progress has been achieved in the management of the disease in recent decades, SLE is still linked to gradual accumulation of irreversible organ damage, which has been demonstrated to predict subsequent damage, increased morbidity burden, and premature death.^3^

To enhance long-term outcomes in SLE, considerable efforts have been dedicated to defining therapeutic goals that linked to improved prognosis for the patients.^4^ Several studies have demonstrated that achieving and maintaining remission in SLE is associated with improved outcomes and extended survival.^5^ However, it has also been observed that even maintaining lupus low disease activity state (LLDAS), with minimal use of glucocorticoids (GCs), can enhance patients’ prognosis and survival; thus, LLDAS is considered an acceptable target for treatment whenever remission cannot be achieved.^6^ Additionally, other important goals encompass early diagnosis, effective and less toxic therapeutic options, appropriate GC tapering, and importantly, the best possible health-related quality of life (HRQoL).^7^ These objectives together contribute to optimising patient outcomes and to improving the overall care of people with SLE.

In this review, we delve into the advancements made in identifying measurable and attainable treatment outcomes in SLE. We emphasise how improved outcome measures and the anticipated arrival of new effective treatments have the potential to prevent disease flares, minimise organ damage, and enhance overall quality of life in people with SLE. We anticipate that these developments will enable the routine implementation of treat-to-target (T2T) approach in the care of these patients.

## RATIONALE FOR A T2T STRATEGY IN SLE

The T2T strategy involves making treatment adjustments with the purpose of attaining a clearly defined and clinically meaningful goal. Those adjustments may be considered at predefined timepoints upon commencement of a new therapy, or at timepoints tailored to the individual patient’s needs. Over the last decades, the concept for management of several prevalent chronic diseases has transitioned from symptom-based to target-based strategies.^8^ This shift has been driven by compelling evidence indicating that target-based approaches lead to improved outcomes. An example of this shift is evident in the treatment of hypertension; by focusing on achieving suitable values for systolic or diastolic blood pressure, significant long-term reductions in the risks associated with cardiovascular diseases have been observed.^9^ Similarly, in the management of diabetes, targeting towards specific blood glucose values measured through haemoglobin A1c has resulted in substantial advancements in patients’ prognosis.^10^

**Figure 1.**
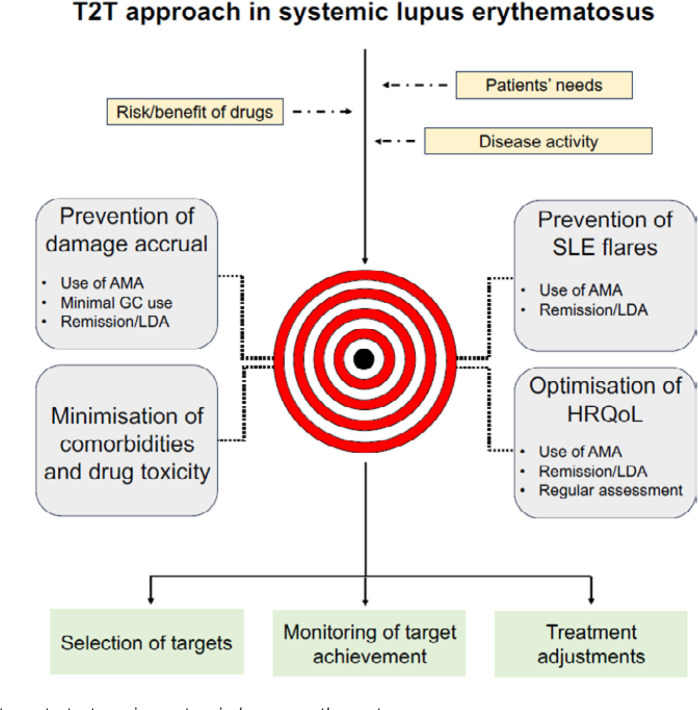
Treat to target strategy in systemic lupus erythematosus. AMA: antimalarial agents; GC: glucocorticoid; HRQoL: health-related quality of life; LDA: low disease activity; SLE: systemic lupus erythematosus; T2T: treat-to-target.

In rheumatic diseases, the goal for therapy differs from the aforementioned conditions in that it often requires simultaneous normalisation or improvement of multiple parameters owing to disease heterogeneity. This aspect adds complexity to T2T approaches, as the definition of the goals is not based on a single parameter but multiple clinical and laboratory features that serve as indicators of disease activity or prognosis.^8^ This, in turn, requires the use of composite measures which incorporate this information and transform it into a dichotomous output. Nevertheless, in the context of rheumatoid arthritis (RA), several randomised clinical trials (RCTs) and observational studies have consistently shown that T2T approaches lead to improved outcomes in terms of disease progression, long-term damage, and functional status.^11–13^ The first T2T RCT applied to RA was the Tight Control in RA (TICORA) trial.^13^ The aim in TICORA was to reduce disease activity scores through monthly assessments and mandatory adjustments in therapy if the target was not achieved. This trial demonstrated that T2T led to improved treatment responses, higher rates of remission, and reduced radiographic damage compared to standard care. Additional studies further supported the benefit of T2T in RA by showing improvements in physical function, HRQoL, and effective prevention of radiographic damage.^14–16^ This evidence has led to the development of T2T recommendations for RA, which have prompted further investigations and implementation of such approaches in routine clinical practice.^17^ Following the example of RA, the importance of T2T approaches has also been recognised in other rheumatic diseases such as spondylarthritis, gout, and psoriatic arthritis.^18–20^ SLE is a more complex disease with multiple facets that require attention for successful management, including the control of disease activity, prevention of damage progression, minimisation of treatment-related side-effects, and enhancement of patients’ quality of life.^21^ It is crucial to have a deep understanding of the lupus natural history, as the ultimate objective is to alter its course. Thus, attainment of the chosen targets of therapy should show ability to exert a clear benefit for the patient by modifying the disease trajectory (**Figure 1**).

## RECOMMENDATIONS FOR T2T IN SLE

To investigate the applicability of the T2T concept in SLE management, an international task force was assembled in 2014.^22^ This international task force formulated recommendations for implementing a T2T approach in SLE, highlighting the need for further advancements to achieve the defined objectives (**Table 1**). Making T2T feasible in clinical practice requires the establishment of practical and achievable outcome measures, as well as the development of therapeutic options that realistically enable the attainment of these targets. The T2T task force identified specific targets, placing particular emphasis on managing disease activity and preventing irreversible organ damage, while aiming to minimise glucocorticoid use and facilitate their withdrawal whenever feasible. Furthermore, the recommendations underscored the importance of considering SLE patients’ HRQoL as a crucial factor in treatment decisions. Over the past decade, substantial progress has been made in achieving these objectives in SLE.^23^ Importantly, evidence has been gathered on outcomes based on clinically meaningful disease activity states, such as the LLDAS.^24,25^ Additionally, the Definition of Remission in SLE (DORIS) task force provided a clear definition of remission as the ultimate treatment goal.^26^ These advancements, coupled with the urgent need for more effective and safer therapies, have led to an unprecedented growth in clinical trials in SLE. Encouraging results have been observed with various novel therapies, including biologics and small-molecule agents, indicating promising avenues for future treatment options in SLE.^27^

**Table 1.** Principles for treat to target in systemic lupus erythematosus.

1. The treatment target of SLE should be remission of systemic symptoms and organ manifestations or, when remission cannot be reached, the lowest possible disease activity, measured by a validated lupus activity index and/or by organ-specific markers.
2. Prevention of flares (especially severe flares) is a realistic target in SLE and should be a therapeutic goal.
3. It is not recommended that the treatment in clinically asymptomatic patients be escalated based solely on stable or persistent serological activity.
4. Since established organ damage predicts subsequent accrual of organ damage and death, prevention of organ damage accrual should be a major therapeutic goal in SLE.
5. Factors negatively influencing health-related quality of life (HRQoL), such as fatigue, pain, and depression should be addressed, in addition to control of disease activity and prevention of organ damage.
6. Early recognition and treatment of renal involvement in lupus patients is strongly recommended.
7. For lupus nephritis, following the initial phase of therapy for induction of remission, at least 3 years of subsequent immunosuppressive treatment is recommended to optimise outcomes.
8. Lupus maintenance treatment should aim for the lowest glucocorticoid dosage needed to control disease, and if possible, glucocorticoids should be withdrawn completely.
9. Prevention and treatment of antiphospholipid syndrome (APS)-related morbidity should be a therapeutic goal in SLE; therapeutic recommendations do not differ from those in primary APS.
10. Irrespective of the use of other treatments, serious consideration should be given to the use of antimalarials, which are recommended in all cases unless a contraindication exists.
11. Relevant therapies adjunctive to immunomodulation or immunosuppression should be considered to control comorbidities in SLE patients.

## TARGETS OF TREATMENT

### Remission and low disease activity

Numerous interpretations of remission have come to light over the past decade (**Table 2**).^5,28–30^ In 2016, discussions were initiated around definition of remission within the frame of the international definition of remission in SLE (DORIS) task force,^5^ which later led to a prevailing definition, published in 2021.^26^ Within the framework of the prevailing DORIS definition, serological activity was deliberately excluded, since no unequivocal linear correlation has been discerned between serological markers (complement and anti-dsDNA levels) and disease activity in SLE.^31^ Follow-up studies showed that attainment of remission bears a profound association with a marked decrease in both disease flare rates and organ damage accrual.^32^ Recent investigations have indicated that durability in this state matters, since prolonged remission directly influences outcomes, including enhanced mitigation of damage accrual and improved HRQoL experience among patients.^32–34^

**Table 2.** Different definitions of remission.

	**Criteria for remission**

van Vollenhoven et al.^8^	cSLEDAI=0
	PGA <0.5
	PDN dose ≤5mg/day
	HCQ, stable IS, biologics allowed

Polachek et al.^9^	cSLEDAI=0
	PDN dose 0 mg/day
	HCQ allowed

Ugarte-Gil et al.^10^	SELENA-SLEDAI=0
	PDN dose ≤5mg/day
	HCQ, stable IS allowed

Zen et al.^7^	cSLEDAI=0
	PDN dose ≤5mg/day
	HCQ, stable IS, biologics allowed

cSLEDAI: clinical SLE disease activity index; HCQ: Hydroxycloroquine; IS: immunosuppressants; PGA: Physician Global Assessment; PDN: prednisone; SELENA-SLEDAI: Systemic Lupus Erythematosus Disease Activity Index.

According to the DORIS task force guidelines, the state of remission is solely attainable when the daily administration of prednisone is equal to or less than 5 mg, in conjunction with steady maintenance doses of immunosuppressive or biologic agents.^5^ It is widely acknowledged that prolonged administration of GCs, even at reduced dosages, can be deleterious, leading to increased damage accumulation.^28^ Hence, as a part of the T2T strategy, cessation of the glucocorticoid therapy should be undertaken as soon as it is practically achievable.^35^

When remission cannot be achieved, low disease activity (LDA) provides a viable objective for disease management.^36^ Among several and diverse definitions for LDA,^37^ the criteria laid out by the Asia Pacific Lupus Collaboration group, together forming the definition of LLDAS, are the most widely used in clinical studies of SLE.^24^ This definition allows a slightly higher daily dose of GCs compared with the DORIS remission, yet not exceeding 7.5 mg (**Table 3**).^5^

**Table 3.** Different definitions of Low Disease Activity (LDA).

	**Criteria for LDA**

Franklyn et al.^24,25^	SLEDAI-2K ≤4
	PGA ≤1
	PDN dose ≤7.5 mg/day
	HCQ, stable IS, biologics allowed

Polachek et al.^30^	cSLEDAI≤2
	PDN dose 0 mg/day
	HCQ allowed

Ugarte-Gil et al.^29^	SELENA-SLEDAI≤4
	PDN dose ≤7.5 mg/day
	HCQ, stable IS, biologics allowed

cSLEDAI: clinical SLE disease activity index; HCQ: Hydroxycloroquine; IS: immunosuppressants; LDA: low disease activity; PGA: Physician Global Assessment; PDN: prednisone; SELENA-SLEDAI: Systemic Lupus Erythematosus Disease Activity Index; SLEDAI-2K: Systemic Lupus Erythematosus Disease Activity Index 2000.

Non-attainment of LLDAS within six months from treatment initiation has been shown to be associated with organ damage accumulation.^38^ Recent studies have divulged that attainment of LLDAS coincides with favourable short-term outcomes,^33,39^ including favourable HRQoL outcomes.^40^ A recent study demonstrated that achievement of DORIS remission and/or LLDAS for more than 6 months is associated with reduced damage accrual (HR=0.58; 95% CI: 0.36–0.93 for DORIS remission and HR=0.61; 95% CI: 0.43–0.86 for LLDAS) and severe flares (HR=0.14; 95% CI: 0.08–0.27 for DORIS remission and HR=0.19; 95% CI: 0.13–0.27 for LLDAS).^41^ Patients who achieved LLDAS but not DORIS remission, experienced more favourable outcomes with respect to damage accrual and flares compared to patients who did not attain any of the targets. More importantly, attainment of either DORIS remission for more than 2 years or LLDAS for more than 3 years, resulted in damage-free progression of the disease. Another prospective study showed that attainment of LLDAS for at least 50% of the follow-up time yielded a reduced probability to flare or accrue organ damage accrual as well as a reduced cardiovascular risk compared.^39^

### Prevention of flares

Prevention of flares, particularly severe flares, is important towards improved prognosis in people with SLE. Hence, stabilisation of the disease and reduction of flare hazards should be considered an independent therapeutic objective, along with aiming for remission or LDA. To date, there exists only sparce evidence concerning the effectiveness of different immunosuppressive agents in protecting against flares in SLE.^42^

Azathioprine has been evaluated in comparison with cyclosporin A in cases of active SLE necessitating a daily prednisolone dose of ≥15 mg, yielding similar outcomes in terms of diminishing disease activity and preventing flare occurrence.^43^ In a randomised controlled trial, individuals with quiescent disease who persisted with hydroxychloroquine (HCQ) treatment exhibited a 74% reduced likelihood of experiencing severe flares in contrast to counterparts who ceased the medication.^44^ This safeguarding influence of HCQ has also been evidenced in patients with stable lupus nephritis.^45^ In cases of active moderate-to-severe lupus, the addition of belimumab alongside standard treatment resulted in a noteworthy 36% reduction in the likelihood of experiencing severe relapses over the course of one year.^46^ Concerning lupus nephritis, a study involving Caucasian patients with proliferative lupus nephritis demonstrated that the persistent administration of azathioprine was comparable in effectiveness to mycophenolate mofetil for averting renal flares and the progression towards end-stage kidney disease throughout a 10-year follow-up.^47^ However, in the ethnically diverse Aspreva Lupus Management Study, the continuation of mycophenolate mofetil as maintenance therapy exhibited a notably lower incidence of renal relapses in comparison to azathioprine, spanning a duration of 3 years.^48^ Achieving an optimal strategy for tapering immunosuppressive drugs is equally important for mitigating the likelihood of SLE flares. An observational analysis in a large lupus cohort revealed that the absence of serological activity coupled with a gradual reduction of the dose of immunosuppressant served as predictive factors for a successful withdrawal of medications without experiencing relapses.^42^ Moreover, extended periods of immunosuppressive treatment and sustained renal response are associated with an enhanced probability of successful drug withdrawal in patients with lupus nephritis. In alignment with these findings, transitioning from mycophenolate to less potent agents like azathioprine or calcineurin inhibitors prior to 2 years post the attainment of renal response has been shown to be associated with an almost 2-fold elevated risk of subsequent flare occurrence.^49,50^ To this end, healthcare practitioners should give particular attention to any instances of non-adherence to medication and evaluate potential underlying factors.^51^ Non-compliance with lupus treatment has been linked to heightened susceptibility to disease relapses and a rise in the utilisation of emergency medical services.^52^

### Prevention of organ damage accrual

In SLE, organ damage seems to occur early during the disease course; up to 40% of patients develop damage within one year from diagnosis.^53^ Since damage is tightly linked to mortality, prevention of damage stands for a major therapeutic goal for SLE patients. The current European League Against Rheumatism (EULAR) recommendations for the management of SLE encompass the treatment goals of preventing organ damage accrual, reducing drug-related adverse events, and reducing the dose of GCs to the lowest possible dose, or withdrawal whenever feasible.^1^ Organ damage can be caused by multiple factors such as persistency of disease activity as well as drug toxicity, especially by GCs and broad immunosuppressants.^54^ Additionally, damage frequently occurs in the cardiovascular and renal systems, which have a strong deleterious impact on survival.^54^ Hence, strategies for preventing organ damage should include control of disease activity and minimisation of GC therapy.^4^ Inability to attain low disease activity within the initial 6 months of diagnosis has been linked to early accumulation of organ damage.^38^ In another interesting study, Ruiz-Arruza et al. compared a conventional treatment approach involving high doses of GCs with an alternative regimen comprising lower doses of GCs, the use of methylprednisolone pulses, early implementation of other immunosuppressants, and strict use of HCQ.^55^ The patient subgroup that was subjected to reduced GC doses exhibited markedly diminished overall damage accrual, particularly in the items related to GCs and cardiovascular disease.

The discussion below focuses on the early utilisation of HCQ and timely commencement of targeted therapies such as belimumab, including their potential capacity to alter the course of the disease and attenuate organ damage accrual.^56^ However, there remains ongoing deliberation concerning the presence of a true therapeutic window during which SLE genuinely exhibits increased responsiveness to disease-modifying interventions.

## CURRENT THERAPEUTIC OPTIONS FOR ACHIEVING THE TARGETS

### Minimising glucocorticoid dose

GCs constitute a cornerstone treatment for SLE, being powerful inductors of remission. Unfortunately, with current management, GCs are frequently needed over long periods of time. For severe lupus, high doses (0.5–1 mg/kg/day orally or pulses of intravenous methylpredniso-lone 500–1000 mg/day) are often required to control the disease during the early acute phase of a flare. However, cohort studies comparing treatment with high versus low dose of GCs in induction treatment for lupus nephritis found similar rates of renal response.^57–60^ Importantly, the undesirable effects that are associated with GC use are usually dose- and time-dependant^61^ and may be exacerbated in patients with SLE due to the common presence of comorbidities, particularly cardiovascular disease.^62^

A clear association between long-term treatment with GCs and damage accrual has been described in several studies. Apostolopoulos et al. showed that damage accrual was significantly more frequent in GC-exposed (42%) versus non-exposed (15%) SLE patients and with time-adjusted mean doses of prednisolone above 4.42 mg/day.^63^ Zen et al. studied 293 SLE patients during a 7-year period of follow-up and observed that damage was higher in those in clinical remission on GCs (p<0.001) compared with those who did not achieve remission and that a cumulative prednisone dose above 180 mg/month was a predictor of damage accrual [OR=3.1; 95% confidence interval (CI) 1.3–7.7], as was the number of flares per year (OR=8.8; 95% CI: 1.7–45.4).^32^

Considering that organ damage in SLE patients is linked to early and elevated morbidity and mortality, it is advisable to implement an individualised gradual reduction plan for medications. The ultimate objective should consistently be the cessation of GCs whenever this is feasible. Nonetheless, maintaining equilibrium between the reduction of GCs to mitigate toxicity and the risk for SLE flares that accompanies this decrease in immunosuppression remains paramount and constitutes the central apprehension for healthcare practitioners. Mathian et al. suggested that prednisone 5 mg/day may be needed to prevent relapses; in this study, patients randomised to low-dose GCs as maintenance therapy exhibited significantly fewer flares compared with the withdrawal group.^64^ However, an important drawback in the design of this study was the abrupt interruption of the GC therapy in the withdrawal group. In real-life patient settings, gradual tapering and discontinuation of GCs has been suggested to be safe when the disease is clinically inactive and in long-term remission or LLDAS.^65–67^

The rituxilup protocol that aimed to evaluate the combination of rituximab and mycophenolate mofetil without oral GCs in active lupus nephritis employed a steroid-free maintenance regimen. This involved an initial treatment with two doses of rituximab 1 g each and intravenous methylprednisolone 500 mg (with a two-week interval), followed by treatment with mycophenolate mofetil alone. Real-life data from 50 LN cases yielded complete renal remission in 52% of patients and partial renal remission in 34% of patients at one year.^68^ Taking these factors into consideration, the most recent update of the EULAR recommendations for the management of SLE sets the goal at a prednisone equivalent dose ≤5 mg/day, and discontinuation whenever feasible.^69^.

### Antimalarial agents

HCQ has been demonstrated to significantly decrease the risk of flares and organ damage accrual.^70^ Akhavan et al. showed that HCQ was independently associated with less damage accrual (OR=0.34; 95% CI 0.132–0.867), as opposed to age and GC therapy which contributed to damage progression; these findings were similar to those by Petri et al., which also show protective effects induced by HCQ use, albeit less significative.^71,72^

Regarding flares, HCQ was also shown to be associated with a lower frequency of flare occurrence (OR=0.22; 95% CI 0.07–0.73), even after the discontinuation of immunosuppressants (OR=0.243; 95% CI 0.070–0.843).^73^ In 2022, a study from the Systemic Lupus International Collaborating Clinics (SLICC) collaboration which included 1460 SLE patients corroborated these results, and concluded that the hazard ratio (HR) for a flare was higher if HCQ was reduced (HR 1.2; 95% CI 1.04–1.38) or discontinued (HR=1.56; 95% CI 1.31–1.86).^74^

Along similar lines, Costedoat-Chalumeau et al. studied the effects of HCQ blood concentration with regard to SLE exacerbations and demonstrated that SLE patients who developed a flare within a 6-month follow-up had lower blood concentrations of HCQ, with the baseline concentration of HCQ being an independent predictor of subsequent disease exacerbations (OR=0.4; 95% CI 0.18–0.85).^75^

In the LUMINA cohort, an increase in survival rates for SLE patients using HCQ was observed compared with patients who did not use HCQ. Importantly, HCQ demonstrated a protective impact on survival with an odds ratio of 0.128 (95% CI 0.054–0.301). This protective effect remained significant after adjusting for factors influencing treatment decisions.^76^

Furthermore, HCQ is an essential drug for pregnant women with SLE, as it has been shown to decrease the risk of flares during pregnancy, although with no proven efficacy regarding pregnancy or foetal outcomes.^77,78^ In women with positive anti-SSA, HCQ has been shown to be important for reducing the risk of neonatal lupus and foetal atrial-ventricular block, resulting in its recommendation by the American College of Rheumatology and the British Society of Rheumatology in their most recent updates (2020 and 2022, respectively).^79–81^

The benefits of HCQ are well studied and include aspects beyond its direct disease-related effects, such as improvement of lipid and glucose levels and an overall decrease of cardiovascular events.^70,82–84^ Jorge et al. showed a benefit from the use of HCQ in preventing cardiovascular events overall (OR=0.86; 95% CI 0.77–0.97) as well as venous thromboembolism in particular (OR=0.74; 95% CI 0.59–0.94). In a Danish cohort of 3036 SLE patients (1551 with cutaneous lupus), there was an inverse association between HCQ and the risk of major adverse cardiovascular events, with an adjusted HR oof 0.67 (95% CI 0.51–0.89).

The widely adopted daily dose of 5 mg/kg remains the current recommendation, and the most recent EULAR guidelines reaffirmed this dose target. Importantly, higher flare rates have been seen with lower doses.

### Immunosuppressive drugs

When a favourable response to HCQ, with or without GCs, is not evident, alternative immunosuppressive approaches are recommended by the EULAR guidelines. These strategies encompass the implementation of biologics (belimumab, anifrolumab) and synthetic immunosuppressants including methotrexate, azathioprine, mycophenolate, and calcineurin inhibitors (voclosporin, tacrolimus, and cyclosporin A). However, to date, these drugs have not shown any disease-modifying properties. It is crucial to administer these medications at the lowest effective dose while carefully monitoring potential adverse effects. Cyclophosphamide, due to its potential toxicity, is typically reserved for situations involving organ or life-threatening manifestations, particularly severe lupus nephritis and neuropsychiatric lupus.^85^

### Biologics

#### Belimumab

In 2011, belimumab was approved as the first biologic agent for SLE. Post-hoc analyses from the initial RCTs showed that belimumab is associated with protection against damage accrual in SLE patients, reduced flare occurrence, and steroid-sparing effects, and several reports from real-life cohorts confirmed these beneficial effects.^86–93^ Urowitz et al. compared patients under belimumab plus standard therapy versus standard therapy alone and showed that belimumab-treated patients exhibited reduction by 61% in the risk of progressing to a higher SDI score (HR=0.39; 95% CI 0.25–0.61) in a real-life cohort.^86^ Two real-world studies demonstrated that patients with active SLE and low damage at baseline had a higher probability of favourable outcomes if treated early with belimumab.^87,93^ Moreover, Gatto et al. observed a significant decrease with belimumab in exacerbation rates compared with the period before the initiation of the biologic agent.^87^

A German cohort that included 102 patients who received belimumab therapy, 42% showed an improvement of at least 50% in overall disease activity at the 6-month follow-up, with a decrease in SELENA-SLEDAI scores accompanied by a reduction in mean doses of GCs.^88^ Similarly, in an American cohort comprising 501 patients, there was an at least 50% improvement in overall clinical response in 48.7% of the patients within a 6-month follow-up along with a reduction in GC doses.^89^ Scheinberg et al. conducted a study with 48 Brazilian patients which corroborated these findings, with a significant decrease in SLEDAI score (12 ± 3.0 to 2.5 ± 2.5) and GC dose (from 30 ± 12.5 mg to 7.5 ± 5.0 mg).^90^ Similar findings were reported by Andreoli et al. on a small cohort of 18 patients with refractory SLE, i.e., reduction in prednisone dose from 66.3 mg/week to 46.9 mg/week after 9 months, with SLEDAI-2K scores improving from 9 to 6.^91^ In a Greek cohort of 188 patients with active SLE, belimumab helped achieve the therapeutical goals LLDAS and DORIS remission (33.5% and 17.8% of patients at the 24-month follow-up, respectively).^92^ Regarding patient-reported outcomes, Parodis et al. reported consistent benefits with belimumab in pain (p < 0.0001), fatigue (p = 0.007) and general health (p < 0.0001) over a 53-month period of follow-up.^93^

van Vollenhoven et al. studied the BLISS trial datasets to identify predictors of treatment efficacy and found that patients with higher disease activity and serological activity are benefited more from belimumab therapy, while long-standing disease and chronic damage may have a negative impact on its clinical efficacy.^94,95^

Benefits have also been observed for patients with active LN. Furie et al. showed in a RCT comprising 448 patients that belimumab as an add-on therapy to conventional immunosuppression with intravenous cyclophosphamide or mycophenolate yielded greater complete renal response frequencies at week 104 compared with placebo (OR=1.6; 95% CI: 1.0–2.3), along with a good safety profile. Flares were also significantly decreased with belimumab versus placebo in the BLISS-LN trial (HR=0.45; 95% CI: 0.28–0.72; P = 0.0008).^96^ This led to the approval of belimumab for patients with active lupus nephritis, on top of standard therapy.^97^ Parodis et al. also observed in a representative sample of 1844 patients that low-dose intravenous belimumab (1 mg/kg monthly) and subcutaneous belimumab (200 mg weekly) were associated with prevention against de novo renal flares (adjusted HR=0.38; 95% CI: 0.20–0.73; *P* = 0.004 and 0.69; 95% CI: 0.54–0.88; *P* = 0.003, respectively),^98^ with similar observations regarding renal relapses in another post-hoc analysis of clinical trial data by Gomez et al., especially when belimumab was administered along with concomitant administration of antimalarial agents.^99^ However, some cases of de novo renal SLE during belimumab therapy have been reported both in real-world^100,101^ and in clinical trial^102^ settings, illustrating the one-size-does-not-fit-all premise and the need for informed and personalised approaches in treatment selection.

#### Anifrolumab

In 2022, anifrolumab, a human IgG1 monoclonal antibody that binds to the type I interferon receptor subunit 1, received approval for the treatment of active SLE on top of standard therapy. Its mechanism impedes the signalling of all type I interferons, which are crucial components in the pathophysiology of SLE.^103^ Notably, the TULIP-2 trial demonstrated that anifrolumab yielded greater frequencies of British Isles Lupus Assessment Group (BILAG)-based Composite Lupus Assessment (BICLA) response, which in turn allowed for a reduction in GC dosages.^103^ In the context of lupus nephritis, while anifrolumab did not meet the primary endpoint in a phase II RCT, beneficial effects were seen with the intensified anifrolumab regimen, which was superior to placebo in inducing complete renal response.^104^

In studies comparing belimumab versus anifrolumab, differing outcomes emerged; Bruce et al. reported a higher SLE-responder index (SRI)-4 response with anifrolumab, whereas Neupane et al. reported similar benefit from the two drugs, though with a slightly higher likelihood of response with belimumab.^105,106^ These divergent findings underscore the necessity for more comprehensive head-to-head studies. Importantly, direct comparison between the RCTs of belimumab and anifrolumab is limited by the different eras of lupus management, thus anticipated substantial differences in the background therapies given to the patients included in these studies.

#### Rituximab

In the context of SLE, rituximab is used off-label but is primarily reserved for cases that are refractory to standard treatments. While observational studies have suggested that rituximab may be effective in managing severe and refractory SLE, potentially allowing for a reduction in glucocorticoid usage, its performance in RCTs has not been consistent, even rather poor. In fact, in RCTs of patients with either renal or extra-renal SLE, rituximab failed to demonstrate superiority over placebo. However, owing to real-world experiences, off-label use of rituximab is recommended for refractory SLE, and the drug recently even received approval by regulatory agencies in Japan for patients who do not respond sufficiently to existing therapies.^107–110^

### Improving quality of life in systemic lupus erythematosus

Assessment of HRQoL is often overlooked in routine clinical practice, but is gradually gaining more attention. In the case of SLE, there is frequently a lack of agreement between physicians and patients with regard to perceived disease activity and concerns.^23^ The main reason for this discrepancy arises from the fact that physicians primarily focus on routine markers and typical signs of inflammation, while patients’ experiences can be influenced by a wide range of physical, mental, and social factors, as well as comorbid conditions, whose impact often is difficult to distinguish from that of lupus.^21,111^

Recent clinical trials of SLE have incorporated various patient-reported outcome measures (PROMs) of HRQoL as secondary endpoints of efficacy. Post-hoc analyses of RCTs of belimumab have shown clinically meaningful improvements in various HRQoL aspects with belimumab treatments,^112,113^ which has also been seen in real-world investigations.^114^ Among factors influencing HRQoL, organ damage appears to have a major impact,^115^ as do comorbidities like fibromyalgia and obesity.^116,117^ Patients on LLDAS or in remission may demonstrate better HRQoL outcomes, emphasising the importance of T2T management strategies for SLE also from this perspective.^118^

Over the past decade, numerous studies have been conducted to evaluate the impact of LLDAS and/or remission on HRQoL aspects in patients with SLE. In two different studies, it was observed that prolonged remission exceeding 5 years was linked to improved HRQoL based on SF-36 and LupusPRO assessments.^34,119^ Similarly, two observational cohort studies investigating the correlation between LLDAS and HRQoL demonstrated an association between LLDAS and enhanced HRQoL using both a generic instrument (SF-36) and a disease-specific instrument (SLEQOL).^120,121^ Another post-hoc analysis of the BLISS-52 and BLISS-76 trials of belimumab revealed that both remission and LLDAS contributed to favourable HRQoL outcomes, especially in physical aspects, in a time-dependent manner.^118^ Importantly, beyond biologics, also use of antimalarial agents appears to be beneficial with regard to HRQoL experience by patients with SLE.^122,123^

## CONCLUSIONS

The development and validation of remission and LLDAS has offered valuable and substantiated treatment goals, facilitating the adoption of T2T strategies in SLE. However, it is important to acknowledge that observational cohort studies have inherent limitations in establishing direct causal relationships between the attainment of remission or LLDAS and enhanced disease outcomes. To address this, interventional trials implementing T2T approaches are imperative. Such trials should compare the escalation of treatment when LLDAS or remission is not achieved, akin to studies conducted in RA.^13^ To this end, the ongoing LUPUS-BEST trial have been designed to address the implementation of T2T strategy with respect to damage accrual and HRQoL.^124^ Apart from evaluating the causal impact on patient outcomes, such trials will also assess the feasibility of implementing LLDAS or remission in clinical practice, including an evaluation of the needs for resources.^125^ Moreover, the global, multi-stakeholder project “Treatment Response Measure for SLE (TRM-SLE) taskforce” is currently ongoing and aims at developing a novel clinical outcome assessment designed specifically for measuring clinically meaningful effects of interventions in patients with SLE.^126^ Along with the currently existing targeted therapies for SLE, novel drugs currently undergoing clinical trials hold the potential to contribute to enhanced attainment of treatment targets (**Table 4**).

**Table 4.** Phase III randomised clinical trials for systemic lupus erythematosus (2023).

**Drug in study**	**Mechanism of action**	**Main indication**	**Primary Outcome**	**Name of the study**
Anifrolumab	Anti-type I interferon receptor monoclonal antibody	Lupus nephritis class III/IV	Complete renal response	IRIS
Cenerimod	Selective S1P1 receptor modulator	Active SLE (moderate to severe)	Change from baseline to month 12 in the modified SLEDAI-2K score	OPUS-2
Dapirolizumab	Anti-CD40L antibody	Active SLE (moderate to severe)	BICLA response	PHOENYCS GO
Deucravacitinib	Tyrosine kinase 2 inhibitor	Active SLE (moderate to severe)	SRI(4) response	POETYK SLE-2
Ianalumab	Anti-BAFF-receptor antibody	Active SLE Lupus nephritis	SRI(4) response Complete renal response	SIRIUS-SLE 2 SIRIUS-LN
Litifilimab	Anti-BDCA2 antibody	Active SLE	SRI(4) response	TOPAZ-2
Obinutuzumab	Anti-CD20 antibody	Lupus nephritis class III/IV Active SLE	SRI(4) response	REGENCY ALLEGORIA
Telitacicept	TACI-Fc fusion protein targeting BLyS and APRIL	Active SLE (moderate to severe)	SRI(4) response	-
Upadacitinib	JAK inhibitor	Active SLE (moderate to severe)	BICLA response	SELECT-SLE

APRIL: a proliferating-inducing ligand; BAFF: B cell activating factor; BDCA2: blood dendritic cell antigen 2; BICLA: British Isles Lupus Assessment Group (BILAG) – Based Composite Lupus Assessment; BLyS: B lymphocyte stimulator; CD: Cluster of Differentiation; CD40L: Ligand of Cluster of Differentiation 40; JAK: Janus Kinase; SLE: Systemic Lupus Erythematosus; SLEDAI-2K: Systemic Lupus Erythematosus Disease Activity Index 2000; SRI-4: Systemic Lupus Erythematosus Responder Index – 4; TACI-Fc: Fusion protein comprising a recombinant transmembrane activator and calcium modulator and cyclophilin ligand interactor (TACI) receptor fused to the fragment crystallisable domain of human IgG.

In conclusion, remission and LLDAS represent distinct and clinically relevant treatment targets that are associated with reduced adverse outcomes, including disease flares and damage accrual, along with improved HRQoL. With additional research, these endpoints have the potential to facilitate the implementation of T2T approaches in routine patient care and provide robust and discriminative outcome measures for use in clinical trials.
